# Intraoperative liver deformation and organ motion caused by ventilation, laparotomy, and pneumoperitoneum in a porcine model for image-guided liver surgery

**DOI:** 10.1007/s00464-023-10612-x

**Published:** 2023-12-26

**Authors:** Philipp A. Wise, Anas A. Preukschas, Emre Özmen, Nadine Bellemann, Tobias Norajitra, Christof M. Sommer, Christian Stock, Arianeb Mehrabi, Beat P. Müller-Stich, Hannes G. Kenngott, Felix Nickel

**Affiliations:** 1 Department of General, Visceral and Transplantation Surgery, Heidelberg University, Im Neuenheimer Feld 420, 69120 Heidelberg, Germany; 2 Department of General, Visceral and Thoracic Surgery, University Medical Center Hamburg–Eppendorf, Martinistraße 52, 20246 Hamburg, Germany; 3 Department of Diagnostic and Interventional Radiology, Heidelberg University, Im Neuenheimer Feld 420, 69120 Heidelberg, Germany; 4 Division of Medical and Biological Informatics, German Cancer Research Center, Im Neuenheimer Feld 280, 69120 Heidelberg, Germany; 5 Institute for Medical Biometry and Informatics, Heidelberg University, Im Neuenheimer Feld 305, 69120 Heidelberg, Germany; 6 Division of Abdominal Surgery, Clarunis–Academic Centre of Gastrointestinal Diseases, St. Clara and University Hospital of Basel, Petersgraben 4, 4051 Basel, Switzerland

**Keywords:** Soft tissue surgery, Image guidance, Liver motion, Mri, Ventilation, Laparoscopic surgery

## Abstract

**Background:**

Image-guidance promises to make complex situations in liver interventions safer. Clinical success is limited by intraoperative organ motion due to ventilation and surgical manipulation. The aim was to assess influence of different ventilatory and operative states on liver motion in an experimental model.

**Methods:**

Liver motion due to ventilation (expiration, middle, and full inspiration) and operative state (native, laparotomy, and pneumoperitoneum) was assessed in a live porcine model (*n* = 10). Computed tomography (CT)-scans were taken for each pig for each possible combination of factors. Liver motion was measured by the vectors between predefined landmarks along the hepatic vein tree between CT scans after image segmentation.

**Results:**

Liver position changed significantly with ventilation. Peripheral regions of the liver showed significantly higher motion (maximal Euclidean motion 17.9 ± 2.7 mm) than central regions (maximal Euclidean motion 12.6 ± 2.1 mm, *p* < 0.001) across all operative states. The total average motion measured 11.6 ± 0.7 mm (*p* < 0.001). Between the operative states, the position of the liver changed the most from native state to pneumoperitoneum (14.6 ± 0.9 mm, *p* < 0.001). From native state to laparotomy comparatively, the displacement averaged 9.8 ± 1.2 mm (*p* < 0.001). With pneumoperitoneum, the breath-dependent liver motion was significantly reduced when compared to other modalities. Liver motion due to ventilation was 7.7 ± 0.6 mm during pneumoperitoneum, 13.9 ± 1.1 mm with laparotomy, and 13.5 ± 1.4 mm in the native state (*p* < 0.001 in all cases).

**Conclusions:**

Ventilation and application of pneumoperitoneum caused significant changes in liver position. Liver motion was reduced but clearly measurable during pneumoperitoneum. Intraoperative guidance/navigation systems should therefore account for ventilation and intraoperative changes of liver position and peripheral deformation.

Computer-based navigation tools are increasingly being developed and evaluated for liver procedures to support intraoperative real-time knowledge of liver anatomy, and control of pathological structures and structures at risk with high precision [1, 2, 3–6]. Especially minimally invasive procedures, which have significant clinical advantages over open liver surgery but also increased complexity [1, 2, 7–11], can benefit from accurate liver motion modeling and navigation [12–14]. The liver, like other abdominal organs, is subject to soft tissue deformation and positional change depending on whether the patient is lying or standing, during ventilation due to the movement of the diaphragm, and when there is a change in the intraabdominal pressure—such as in the case of pneumoperitoneum and laparotomy [13–25]. Hepatic motion is also an obstacle for many percutaneous treatment modalities, including but not limited to percutaneous biliary drainage, radiotherapy [15, 16], chemoembolization [26], and percutaneous or transjugular needle biopsy, cryotherapy [13], and microwave ablation [27]. This creates considerable difficulties for surgical computer assistance systems—the most important one being the discrepancy between the preoperative computer model and the intraoperative reality due to soft tissue deformation [28–30]. In order to compensate for the differences in liver position pre- and intraoperatively, an accurate model of the liver motion is needed [1, 2, 13, 15, 16, 26, 27]. Image-guided methods are being researched for intraoperative navigation and image acquisition, which must also take into account the motion and deformation of the liver [31–35].

To create such a model, it is indispensable to analyze the liver motion and deformation. The porcine liver is suitable to analyze such liver changes due to its similarity to that of humans [36]. Several methods for tracking the liver motion have been reported in the literature such as markers [37], contour tracking [17], center of gravity [16, 18], tumor center of mass [19], or vessel tracking [38]. In this study, tracking was performed using the positions of predefined branching points of the hepatic veins and their tributaries. This method is able to show different amounts of movement in different parts of the liver [38–42]. There are a number of studies on liver shift, most of them focusing on the effect of ventilation, however without taking into account different breathing volumes [13–22]. Differences in liver motion according to the operative state have been assessed [24]; however, the current study appears to be the first which attempts to account for the combination of ventilatory volume and operative state.

The aim of the present study was therefore to assess effects of ventilatory volume, pneumoperitoneum, and laparotomy on liver motion in order to improve surgical navigation.

## Materials and methods

### Subjects

A porcine model was used (*n* = 10, German landrace, 20–34 kg). The professional care and handling of animals were carried out by the staff of the Interfaculty Biomedical Research Facility at Heidelberg University. The study protocol was approved by the local Ethics Committee Heidelberg (A 19/08) [25]. The animals were fasted 12 h before the intervention. Premedication was done with azaperone [0.1 mg/kg], midazolam [0.1 mg/kg], and ketamine [15 mg/kg]. The induction of anesthesia was then carried out by intravenous midazolam [0.1 mg/kg] and ketamine [20 mg/kg]. Anesthesia was maintained with intravenous midazolam [0.05 mg/kg], ketamine [10 mg/kg], and pancuronium as needed. The animals were mechanically ventilated (frequency = 12/min, ventilation volume = 8–10 ml/kg). The animals were positioned in a 0° supine position on a vacuum mattress which was firmly attached to a stretcher. This guaranteed full immobilization of the animals during the procedure, so liver motion due to repositioning of the animal did not need to be accounted for [43]. The animals on the stretcher were stabilized and fixed on the CT scan table for the entire duration of the experiments to minimize repositioning errors. The animals were under general anesthesia with machine ventilation during the entirety of experiments and at the end of the procedures they were euthanized using intravenous potassium chloride [150 mg/kg].

### Imaging

Each pig was examined by Computed Tomography as indicated in Table 1. CT scans were taken with a slice thickness of 2 mm with 1 mm overlay (SOMATOM Sensation™, 64 Row Dual Energy, Siemens Corp., Erlangen, Germany). In each animal, they were obtained for three ventilatory states (full expiration, middle inspiration, and deep inspiration) in each of the three operative states (native, pneumoperitoneum, and laparotomy), which add up to nine CT scans for each animal. CT scans were performed after creating and maintaining predetermined manually controlled breath-hold positions for each ventilatory state. High tidal volume was defined as 14 ml/kg (deep inspiration, approximately 400 ml) and middle tidal volume as 7 ml/kg (normal inspiration, approximately 200 ml). These definitions of lung volumes were based on previous findings in [39, 44, 45]. Pneumoperitoneum was created and maintained at 15 mmHg of pressure using a standard Veress-needle in the left lower quadrant of the abdomen and a standard pressure-controlled insufflation device.

**Table 1:** CT protocol indicating sequence of imaging acquisition with count of CT images per acquisition state

	Native		Pneumoperitoneum		Laparotomy		Total
Expiration	1 Native-Exp	10	4 Pneu-Exp	10	7 Lap-Exp	10	30
Inspiration 200 ml	2 Native-Insp 200	10	5 Pneu-Insp 200	10	8 Lap-Insp 200	10	30
Inspiration 400 ml	3 Native-Insp 400	10	6 Pneu-Insp 400	10	9 Lap-Insp 400	10	30
	30		30		30		180

After imaging with pneumoperitoneum was complete, the Veress-needle was removed and a standard midline laparotomy was performed. The data from the CT scans were transferred onto a mobile hard disk using the Digital Imaging and Communications in Medicine (DICOM) standard.

### Liver segmentation

Obtained CT scans were post-processed using the Medical Imaging Interaction Toolbox (MITK) (www.mitk.org), developed by Division of Medical and Biological Informatics at the German Cancer Research Center (DKFZ) in Heidelberg. For segmentation, the hepatic veins were identified in CT scans and seed points inside the lumens were manually selected for region growing. The minimum and maximum intensity thresholds were adjusted for the region growing algorithm. The thresholds that resulted in the best region growing were found out by trial-and-error for each scan. All segmentations and measurements were cross-checked by at least one experienced radiologist to minimize inter- and intra-user variability.

### Three-dimensional modeling and point mapping

**Fig. 1:**
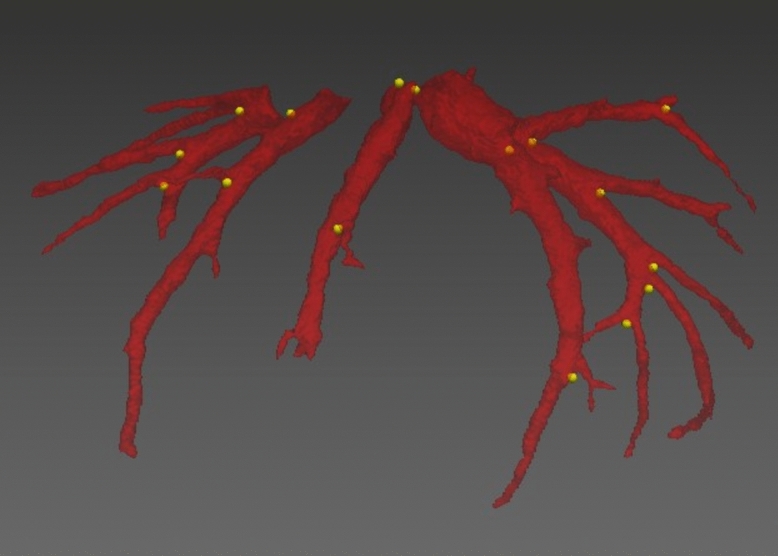
Hepatic veins (red) and marked bifurcation points used for motion analysis (yellow) (Color figure online)

**Fig. 2:**
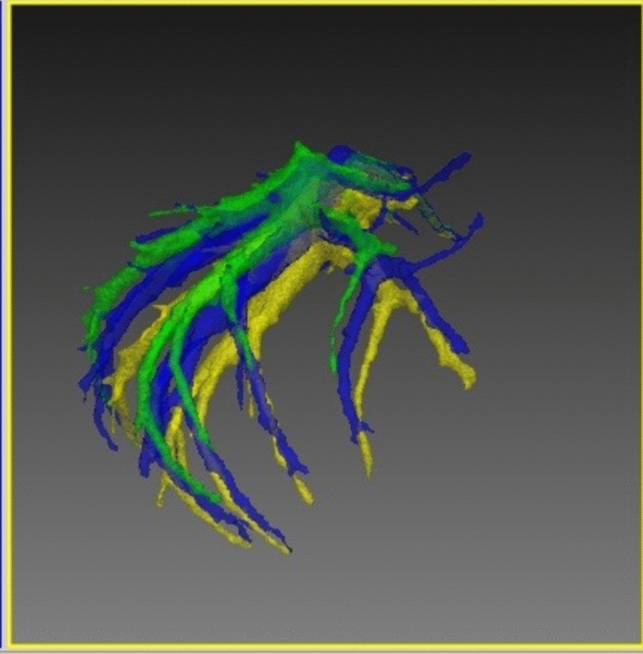
Hepatic veins in full expiration (green), middle inspiration (blue), and full inspiration (yellow) (Color figure online)

The segmented hepatic veins were converted to 3D polygonal models of the hepatic vein tree using the MITK software. Bifurcation points were used for point mapping, rather than center of vessel lumens because of the potential for achieving a greater consistency with the tools used. The first step was to determine the common bifurcation points within each pig separately, which adequately represented the central to peripheral range of the models. Points were mapped using the transverse, sagittal, and coronal views and were confirmed on the 3D models (Fig. 1). The amount of bifurcation points varied between pigs (7–16 points). Rare trifurcations were treated as bifurcations between the two largest of the three branches. The consistency of the points was then re-examined by an experienced radiologist. The use of branching points allowed reliable measurement of liver motion in different locations [42] (Fig. 2).

### Statistical analysis

Statistical analysis was performed with the R programming language (R Foundation for Statistical Computing, Vienna, Austria). Standard packages and additionally the lme4 (Linear mixed-effects models using S4 classes), lmer (Tests for random and fixed effects for linear mixed effect models), and car (Companion to Applied Regression) packages were used. Liver motion was indicated as mean ± confidence interval along the lateral, craniocaudal, and ventrodorsal axes as well as Euclidian distance in millimeters, if not otherwise specified. Significance level was set to *α* = 5% two-sided.

### Euclidean distance in surgical context

In the current study, Euclidean distance is utilized to quantify liver movement, calculated as the vector sum of the liver’s movement in three dimensions: craniocaudal (head-to-tail), ventrodorsal (front-to-back), and lateral (side-to-side). The vector sum is the aggregate of movements along each of these anatomical axes, culminating in a single straight-line distance that encapsulates the total displacement of the liver. This approach enables a precise and comprehensive assessment of liver movement in three-dimensional space.

## Results

### Operative states

**Fig. 3:**
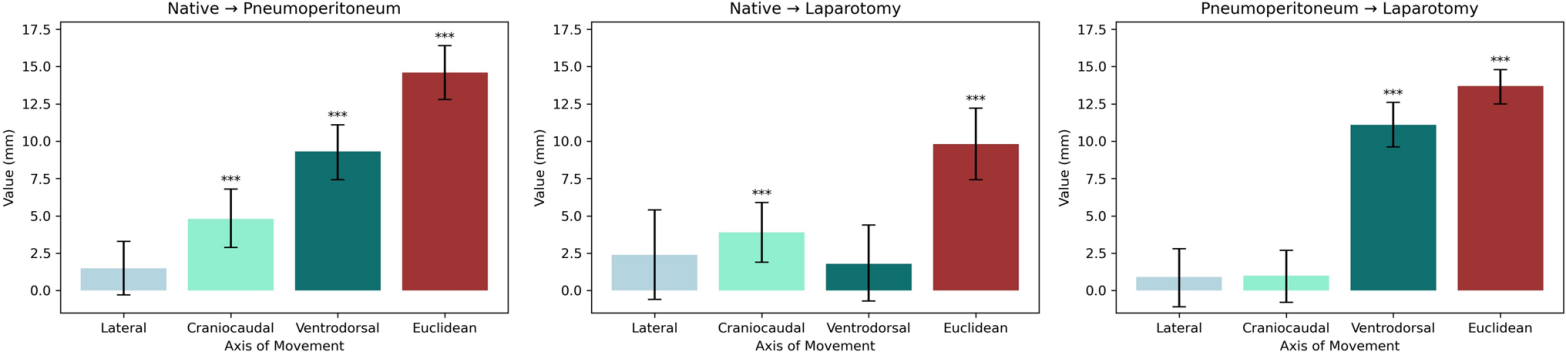
Liver motion between different operative states. Significant motion axes signified as follows: *p* < 0.05 marked with “*,” *p* < 0.01 marked with “**,” and *p* < 0.001 marked with “***”

An overview of the results is seen in Fig. 3. Liver motion between the operative states was analyzed as difference between the arithmetic means of the ventilatory states for each operative state. Liver motion when transitioning between operative states was significant for Euclidian distance in all cases, i.e., native state to pneumoperitoneum, native state to laparotomy, and laparotomy to pneumoperitoneum. The highest amount of motion could be observed along the ventrodorsal and craniocaudal axes. Motion along the craniocaudal axis was significant when transitioning between native state and pneumoperitoneum, as well as between laparotomy and pneumoperitoneum. Motion along the ventrodorsal axis was significant between native state and pneumoperitoneum, as well as native state and laparotomy. No significant motion on the lateral axis could be observed in any transition between operative states.

**Fig. 4:**
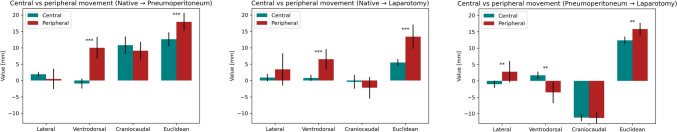
Liver motion between different operative states, stratified by central vein motion vs peripheral vein motion. Significant differences between central and peripheral motion signified as follows: *p* < 0.05 marked with “*,” *p* < 0.01 marked with “**,” and *p* < 0.001 marked with “***”

Figure 4 shows the analysis of liver motion in the different operative states as stratified by location of the measuring points. Both central and peripheral parts of the liver moved significantly with respect to Euclidian distance between all operative states.

Comparing the central and peripheral motion, peripheral parts of the liver moved significantly more than central parts on the ventrodorsal axis and over measured Euclidian distance. Difference in motion was not significant along the lateral axis except when transitioning between pneumoperitoneum and laparotomy, which showed significantly greater peripheral than central movement.

### Ventilatory states

**Fig. 5:**
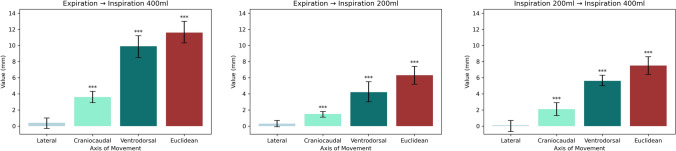
Liver motion between different ventilatory states. Significant motion axes signified as follows: *p* < 0.05 marked with “*,” *p* < 0.01 marked with “**,” and *p* < 0.001 marked with “***”

An overview of the motion stratified by ventilatory state is viewed in Fig. 5. Liver motion between the ventilatory states was analyzed as difference between the arithmetic means of the operative states for each ventilatory state. Motion when transitioning between ventilatory states was significant for the ventrodorsal and craniocaudal axis as well as the Euclidian distance in all operative states. Motion was not significant for the lateral axis. The greatest extent of motion could be observed on the craniocaudal axis. The full extent of motion from full expiration to full inspiration was almost equally distributed between expiration to middle inspiration and from middle inspiration to full inspiration, with slightly more motion observed from middle inspiration to full inspiration.

**Fig. 6:**
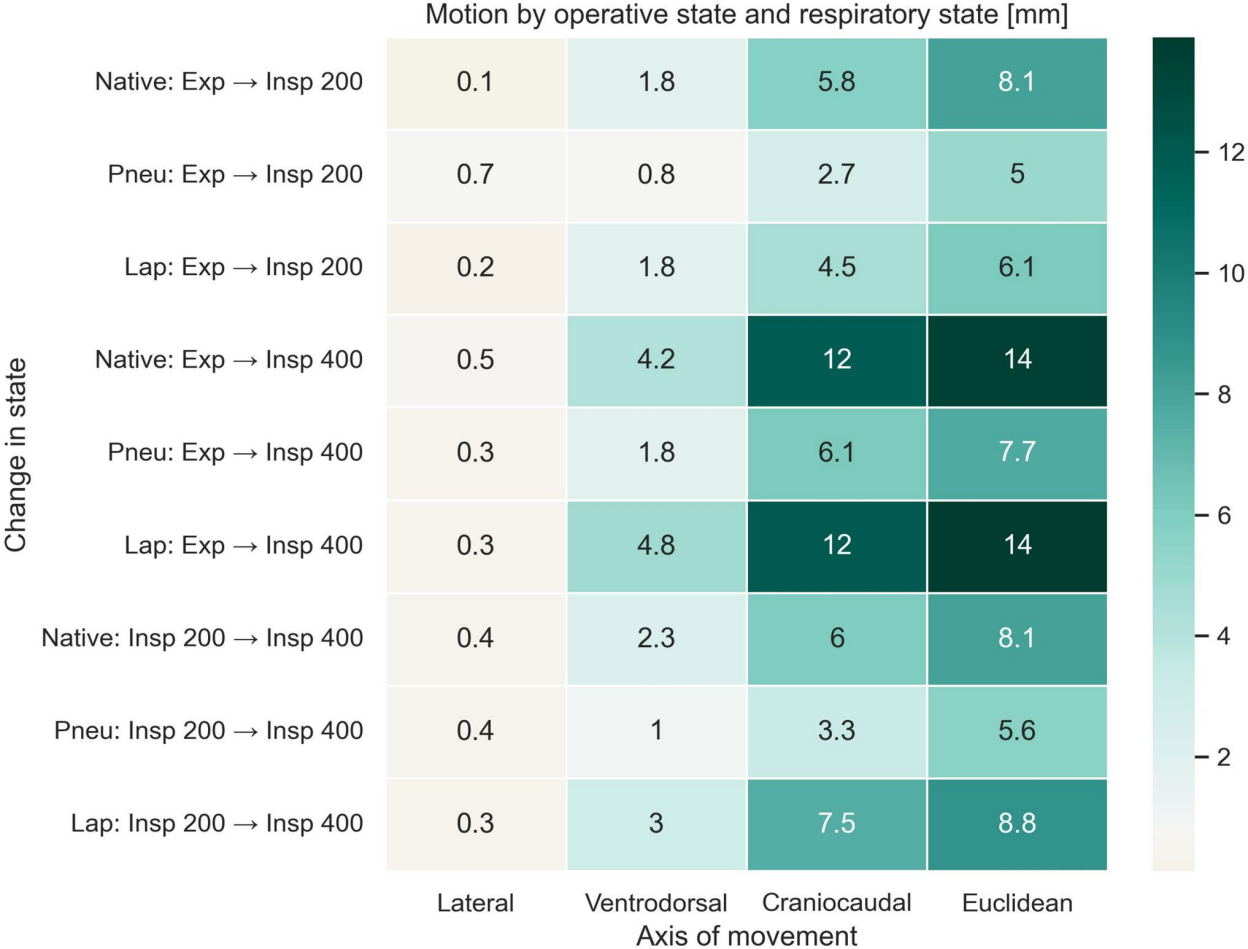
Liver motion between different operative and ventilatory states. Motion is significant on the ventrodorsal and craniocaudal axis as well as Euclidean distance across all subgroups. Motion on the lateral axis is not significant for any subgroup

Figure 6 shows the results of the motion stratified by ventilatory state and operative state. There was significant motion in all subgroups on the craniocaudal axis, ventrodorsal axis, and the Euclidian distance. Motion along the lateral axis was not significant for any subgroup. The greatest extent of motion was observed along the craniocaudal axis. In the native operative state, the full extent of motion from full expiration to full inspiration was equally distributed between full expiration to middle inspiration and middle inspiration to full inspiration. With pneumoperitoneum and laparotomy, there was greater motion from middle inspiration to full inspiration than from full expiration to middle inspiration.

Total liver motion due to ventilation was significantly greater during native and laparotomy operative states comparative to pneumoperitoneum along the ventrodorsal and craniocaudal axis as well as for Euclidian distance. There was no significant motion on the lateral axis when comparing the effect of ventilatory motion on the liver motion between the operative states. Laparotomy did not significantly influence the ventilatory motion of the liver when compared to the native operative state.

## Discussion

The present study demonstrates that ventilation, pneumoperitoneum, and laparotomy caused substantial and distinct liver deformation and liver motion, whereby the peripheral segments of the liver exhibited a greater degree of displacement relative to the central segments. Furthermore, ventilatory volume significantly impacted liver motion across all surgical conditions, albeit to a lesser extent under pneumoperitoneum. Craniocaudal motion emerged as the most pronounced, followed by ventrodorsal motion, while the least motion was observed in the lateral plane.

These findings provide valuable insights into the dynamic behavior of the liver in response to surgical interventions and respiratory dynamics. The current study showed that the highest amount of liver motion as caused by ventilation was evident along the craniocaudal and ventrodorsal axis. These results are in line with studies from human respiratory liver motion. Table 2 gives an overview of publications analyzing ventilation and respiration-related liver motion. In the current study, no significant lateral motion could be observed for any ventilatory change or any operative state transition. Srimathveeravalli et al. also reported that lateral motion contributed least to overall liver motion [39]. The current study supports the argument that lateral motion is less relevant for overall liver motion and deformation.

**Table 2:** Summary of liver motion secondary to ventilation as reported in the literature

Group	Year	Craniocaudal (mm), Normal Inspiration	Craniocaudal (mm), Deep Inspiration	Ventrodorsal (mm)	Lateral (mm)
Suramo et al. [46]	1984	25.0	55.0		
Korin et al. [17]	1992	13.0	39.0		
Davies et al. [21]	1994	10.0 ± 8.0	37.0 ± 8.0		
Kubo et al. [56]	1996	15.0–20.0			
Balter et al. [16]	1996	17.0			
Herline et al. [53]	1999	10.3 ± 2.5			
Shimizu et al. [19]	1999	21.0		8.0	9.0
Shimizu et al. [15]	2000	10.6 ± 7.0		4.6 ± 1.6	5.2 ± 1.8
Brock et al. [57]	2003	8.5		3.8	1.8
Bussels et al. [18]	2003	24.4 ± 16.4		9.0 ± 3.5	13.2 ± 6.9
Rohlfing et al. [22]	2004	12.0–26.0		1.0–12.0	1.0–3.0
Brandner et al. [20]	2006	13.0		5.2	2.1
Beddar et al. [58]	2007	7.5–17.5		1.2–8.7	1.1–5.0
Xi et al. [59]	2009	10.1 ± 3.9		1.2 ± 1.0	1.3 ± 0.5
Nguyen et al. [60]	2009	12.4 ± 1.8		8.4 ± 1.3	1.2 ± 1.0
Brix et al. [40]	2014	11.0		2.5	1.6

There are direct clinical applications that could be considered from the results of this study as well as considerations for navigation and image-guided surgical assistance tools. Image-guided techniques could, for example, be combined with minimally invasive surgery visualization techniques, as well as intraoperative imaging. Combining preoperatively registered images with live feed data could provide information to the surgeon on the displacement of different parts of the liver during various stages of the surgery, allowing for more accurate visualization or localization of, e.g., intrahepatic lesions requiring resection [29, 30]. Additionally, the surgeon may choose to adjust the positioning of the patient or instruments to minimize the displacement of a specific area of the liver that is particularly susceptible to motion, which could help prevent unintended damage to the liver or surrounding structures. Recently, Pelanis et al. investigated the potential of a novel surgical navigation solution [29]. This approach integrated a robotic C-arm for preoperative image data registration and intraoperative updates with fluoroscopic images, achieving a median accuracy of approximately four mm in the published study. The relatively low margin of error is in accordance with the current findings that the least breath-dependent motion was observed during the pneumoperitoneum state. The similar findings also emphasize the dynamic nature of liver motion during surgery and the concomitant necessity of accommodating this aspect within surgical navigation, as the aim of minimally invasive surgery is to minimize resection volume while also avoiding positive resection margins.

There was significant liver motion when switching ventilatory states. This ventilatory-associated motion was observable across all operative states. Earlier studies have shown that craniocaudal motion increased with deep inspiration when compared to normal inspiration [17, 21, 46]. Srimatheveeravalli et al. [39] argued for a linear correlation of tidal volume and liver displacement. Carrying out image-guided procedures with low inspiratory volumes, where it is safe, might reduce liver motion but still causes the liver to move significantly.

In the current investigation, a pneumoperitoneum pressure of 15 mmHg was chosen. This pressure is toward the upper level commonly employed in laparoscopic surgeries, allowing a relevant and realistic differentiation from the other operative states analyzed in the current study. Additionally, existing veterinary literature on pneumoperitoneum shows 15 mmHg to be a commonly utilized pressure in the porcine model [47, 48]. However, acknowledging the potential benefits of understanding the effects of varying pressures on organ deformation and motion, future studies may delve into the impact of different pneumoperitoneal pressures, particularly considering the increasing interest in low pressure pneumoperitoneum. With pneumoperitoneum, liver motion was nearly halved in the current study compared to the native and laparotomy states. This could mean that with laparoscopic surgery, ventilatory motion (< 10 mm) could be reduced for liver navigation. However, by creating the pneumoperitoneum there is considerable liver displacement per se, potentially rendering preoperatively acquired CT images inaccurate without adaption for intraoperative image guidance and navigation. The results of the current study may provide some guidance for minimizing the potential error, as the change from native state to pneumoperitoneum showed consistent, predictable liver displacement. Further research would be required to confirm this motion in human patients. Ventilator settings could then be set to low tidal volume to further reduce potential margin of error, as there was only significant motion along the craniocaudal axis with low tidal volume in the pneumoperitoneum state. Additional optimization could be achieved via intraoperative imaging techniques, such as ultrasound or cone-beam CT, with the patient already in pneumoperitoneum state, which could provide high-resolution images for navigation. Research of the relevant current literature found no other study examining ventilatory liver motion with pneumoperitoneum or laparotomy. The changes in lung and ventilation mechanics under pneumoperitoneum are well documented, including basal atelectasis, compression of the lung basis, and a raised diaphragmatic dome [49–52]. A potential explanation for this is that the physiological and mechanical changes during pneumoperitoneum cause this significant decrease in liver motion due to decreased diaphragmatic motion, which has been shown to be highly correlated to liver motion [53]. Earlier studies report on liver motion due to introduction of pneumoperitoneum. Zijlmans et al. [24] reported liver motion due to pneumoperitoneum with craniocaudal motion of 28.5 ± 1.9 mm, ventrodorsal motion of 20.6 ± 1.8 mm, and lateral motion of 2.5 ± 0.5 mm. Overall motion was 35.3 ± 1.3 mm. Herline et al. [54] reported liver motion due to pneumoperitoneum with craniocaudal motion of 0.1 ± 0.4 mm, ventrodorsal motion of 4.1 ± 6.4 mm, and lateral motion of 1.8 ± 12.0 mm. Overall motion was 2.5 ± 1.4 mm. Vijayan et al. [55] reported of liver motion of up to 44.6 mm with pneumoperitoneum and ventilation. The results of the current study support the findings of Zijlmans et al. with most of the motion along the craniocaudal and ventrodorsal axis and least motion laterally. These results differ from those of Herline et al. The difference to Herline et al. results could be due to different measuring techniques (laparotomy was performed for marker placement) and variations in anesthesia. Heizmann et al. [56] reported intraoperative liver motion of up to 60 mm but did not carry out any measurement to exactly quantify the liver displacement.

There was significant liver motion when the subjects were converted from native state to laparotomy in the present study. This motion was mostly along the ventrodorsal axis and was more pronounced in the peripheral parts of the liver. This may be explained through the incision decreasing the compressive effect of the abdominal wall, thus resulting in increased liver motion. Peripheral parts of the liver moved more than central parts. The liver is partly fixed to the diaphragm by the coronary ligament, which may reduce the motion in the parts close to the inferior vena cava. Furthermore, the differences in the segmental nature of the porcine liver, which has five rather loosely attached lobes, might account for increased peripheral motion [36].

These findings may have clinical implications for image-guided surgical procedures. Enhanced peripheral liver movement underscores the need for real-time adjustments in surgeries such as tumor resections or targeted biopsies, and development of image-guided systems must consider the necessity of accounting for this variance. In the interim, navigation for image-guided interventions might be more suitable for central lesions.

### Limitations

The present study was conducted in an animal model. The porcine liver is similar but not identical to the human liver. The porcine liver has five rather loosely attached lobes, which leaves more room for motion than the two main lobes in the human [36]. Great care needs to be given when transferring the results of this study into a clinical environment. Srimathveeravalli et al. reported on lobe-specific liver motion due to animal positioning and ventilation [39]. Liver motion secondary to repositioning could be neglected, as the animals were immobilized. However, sometimes during an operation, especially with laparoscopic surgery, different positions of the operating table are needed (i.e., Trendelenburg, Anti-Trendelenburg, tilt, etc.). Liver motion due to such repositioning maneuvers would have to be considered. As stated above, the segmental anatomy of the porcine liver is different to the human liver, so that the lobes may move more. Further research needs to be done to clarify if there is significant lobe-specific liver motion in the human liver. Additionally, the porcine model cannot account for the falciform ligament present in humans. This anatomical difference could further influence liver deformation during surgery. Future human-focused studies should investigate the ligament’s impact on liver mobility to better inform intraoperative guidance systems. Objectively identifying the lung volumes of each pig was not feasible within the current research framework, so that lung volume estimation was used as an approximation. Srimathveeravalli et al. [39] defined tidal volumes of 215, 440, and 650 ml as low, middle, and high, respectively. The pigs used were heavier than the ones used in the current study; therefore, lower values were defined in the current study. Physiological tidal volume for pigs is around 7 ml/kg. Lung protective ventilation is < 7 ml/kg and high tidal volume is > 12 ml/kg [39, 44, 45]. 7 and 14 ml/kg were defined as low and high tidal volume the present study, which approximated to 200 and 400 ml, respectively. This falls short of an objective measurement of exact inspiratory volumes, and should be acknowledged as a weakness in this study.

In the current investigation, the effects of varying positive end-expiratory pressure (PEEP) levels on liver motion and deformation were not explicitly explored, as the stated aim of the study was designed to assess liver motion under a range of ventilatory and operative conditions. However, it is important to note that the pressure ranges typically experienced during PEEP are likely to lie within the maximal inspiration and expiration pressures analyzed in our study. Therefore, while not directly assessed, the impact of PEEP on liver motion may be inferred to some extent from our findings. Further detailed examination of PEEP’s specific effects on liver dynamics could offer valuable insights, potentially augmenting the current understanding of liver motion in surgical contexts.

Vascular segmentations cannot be objectively validated as there is no gold standard to compare it to [57]. A widely used method to evaluate liver motion is to compare the mean distance between branching points/anatomical landmarks identified by experts [42]. This manual input should be acknowledged as a potential source of error.

In summary, the current study observed significant variations in liver positioning and motion under various conditions, notably identifying more pronounced movement in the peripheral regions compared to the central areas. Both ventilation and surgical access (Pneumoperitoneum and laparotomy) showed significant influence on the motion and deformation of the liver. This motion was decreased with low ventilation volumes. There was less liver motion due to ventilation with pneumoperitoneum than in native and laparotomy states.

Moreover, the variability in liver motion across different ventilatory and operative states, as highlighted by the current study, emphasizes the necessity for adaptable surgical planning. Surgical imaging systems informed by these data could tailor their strategies based on the liver’s specific state, potentially reducing risks associated with liver mobility and offering a more personalized approach to liver surgery.

The current study provides novel insights into three-dimensional liver motion during ventilation, analyzing both ventilatory and operative states. This research aims to enhance understanding of liver and vessel dynamics, potentially demonstrating initial feasibility and informing the development of more adaptable surgical navigation systems. These findings may aid in enhancing the precision of both intraoperative and endovascular guidance, with the aim of improving surgical accuracy and patient outcomes.

## References

[1] Lamade W, Vetter M, Hassenpflug P, Thorn M, Meinzer HP, Herfarth C (2002). Navigation and image-guided HBP surgery: a review and preview. J Hepatobiliary Pancreat Surg.

[2] Grenacher L, Thorn M, Knaebel HP, Vetter M, Hassenpflug P, Kraus T, Meinzer HP, Buchler MW, Kauffmann GW, Richter GM (2005). The role of 3-D imaging and computer-based postprocessing for surgery of the liver and pancreas. Rofo.

[3] Banz VM, Baechtold M, Weber S, Peterhans M, Inderbitzin D, Candinas D (2014). Computer planned, image-guided combined resection and ablation for bilobar colorectal liver metastases. World J Gastroenterol.

[4] Donati M, Basile F, Stavrou GA, Oldhafer KJ (2013). Navigation systems in liver surgery: the new challenge for surgical research. J Laparoendosc Adv Surg Tech A.

[5] Kenngott HG, Wagner M, Gondan M, Nickel F, Nolden M, Fetzer A, Weitz J, Fischer L, Speidel S, Meinzer HP, Bockler D, Buchler MW, Muller-Stich BP (2014). Real-time image guidance in laparoscopic liver surgery: first clinical experience with a guidance system based on intraoperative CT imaging. Surg Endosc.

[6] Kenngott HG, Wagner M, Nickel F, Wekerle AL, Preukschas A, Apitz M, Schulte T, Rempel R, Mietkowski P, Wagner F, Termer A, Muller-Stich BP (2015). Computer-assisted abdominal surgery: new technologies. Langenbecks Arch Surg.

[7] Troisi R, Montalti R, Smeets P, Van Huysse J, Van Vlierberghe H, Colle I, De Gendt S, de Hemptinne B (2008). The value of laparoscopic liver surgery for solid benign hepatic tumors. Surg Endosc.

[8] Jackson NR, Hauch A, Hu T, Buell JF, Slakey DP, Kandil E (2015). The safety and efficacy of approaches to liver resection: a meta-analysis. J Soc Laprosc Robot Surg.

[9] Slakey DP, Simms E, Drew B, Yazdi F, Roberts B (2013). Complications of liver resection: laparoscopic versus open procedures. J Soc Laprosc Robot Surg.

[10] Buell JF, Cherqui D, Geller DA, O’Rourke N, Iannitti D, Dagher I, Koffron AJ, Thomas M, Gayet B, Han HS, Wakabayashi G, Belli G, Kaneko H, Ker CG, Scatton O, Laurent A, Abdalla EK, Chaudhury P, Dutson E, Gamblin C, D’Angelica M, Nagorney D, Testa G, Labow D, Manas D, Poon RT, Nelson H, Martin R, Clary B, Pinson WC, Martinie J, Vauthey JN, Goldstein R, Roayaie S, Barlet D, Espat J, Abecassis M, Rees M, Fong Y, McMasters KM, Broelsch C, Busuttil R, Belghiti J, Strasberg S, Chari RS (2009). The international position on laparoscopic liver surgery: the Louisville statement, 2008. Ann Surg.

[11] Wakabayashi G, Cherqui D, Geller DA, Buell JF, Kaneko H, Han HS, Asbun H, O’Rourke N, Tanabe M, Koffron AJ, Tsung A, Soubrane O, Machado MA, Gayet B, Troisi RI, Pessaux P, Van Dam RM, Scatton O, Abu Hilal M, Belli G, Kwon CH, Edwin B, Choi GH, Aldrighetti LA, Cai X, Cleary S, Chen KH, Schon MR, Sugioka A, Tang CN, Herman P, Pekolj J, Chen XP, Dagher I, Jarnagin W, Yamamoto M, Strong R, Jagannath P, Lo CM, Clavien PA, Kokudo N, Barkun J, Strasberg SM (2015). Recommendations for laparoscopic liver resection: a report from the second international consensus conference held in Morioka. Ann Surg.

[12] Mutter D, Dallemagne B, Bailey C, Soler L, Marescaux J (2009). 3D virtual reality and selective vascular control for laparoscopic left hepatic lobectomy. Surg Endosc.

[13] Clifford MA, Banovac F, Levy E, Cleary K (2002). Assessment of hepatic motion secondary to respiration for computer assisted interventions. Comput Aided Surg.

[14] Mise Y, Tani K, Aoki T, Sakamoto Y, Hasegawa K, Sugawara Y, Kokudo N (2013). Virtual liver resection: computer-assisted operation planning using a three-dimensional liver representation. J Hepatobiliary Pancreat Sci.

[15] Shimizu S, Shirato H, Aoyama H, Hashimoto S, Nishioka T, Yamazaki A, Kagei K, Miyasaka K (2000). High-speed magnetic resonance imaging for four-dimensional treatment planning of conformal radiotherapy of moving body tumors. Int J Radiat Oncol Biol Phys.

[16] Balter JM, Ten Haken RK, Lawrence TS, Lam KL, Robertson JM (1996). Uncertainties in CT-based radiation therapy treatment planning associated with patient breathing. Int J Radiat Oncol Biol Phys.

[17] Korin HW, Ehman RL, Riederer SJ, Felmlee JP, Grimm RC (1992). Respiratory kinematics of the upper abdominal organs: a quantitative study. Magn Reson Med.

[18] Bussels B, Goethals L, Feron M, Bielen D, Dymarkowski S, Suetens P, Haustermans K (2003). Respiration-induced movement of the upper abdominal organs: a pitfall for the three-dimensional conformal radiation treatment of pancreatic cancer. Radiother Oncol.

[19] Shimizu S, Shirato H, Xo B, Kagei K, Nishioka T, Hashimoto S, Tsuchiya K, Aoyama H, Miyasaka K (1999). Three-dimensional movement of a liver tumor detected by high-speed magnetic resonance imaging. Radiother Oncol.

[20] Brandner ED, Wu A, Chen H, Heron D, Kalnicki S, Komanduri K, Gerszten K, Burton S, Ahmed I, Shou Z (2006). Abdominal organ motion measured using 4D CT. Int J Radiat Oncol Biol Phys.

[21] Davies SC, Hill AL, Holmes RB, Halliwell M, Jackson PC (1994). Ultrasound quantitation of respiratory organ motion in the upper abdomen. Br J Radiol.

[22] Rohlfing T, Maurer CR, Jr., O’Dell WG, Zhong J,  (2004). Modeling liver motion and deformation during the respiratory cycle using intensity-based nonrigid registration of gated MR images. Med Phys.

[23] Moyano-Cuevas JL, Sanchez-Margallo FM, Maestre-Antequera J, Davila-Gomez L, Pagador JB, Sanchez-Peralta LF, Latorre R (2012). Effects of pneumoperitoneum and body position on the morphology of abdominal vascular structures analyzed in MRI. J Magn Reson Imaging.

[24] Zijlmans M, Lango T, Hofstad EF, Van Swol CF, Rethy A (2012). Navigated laparoscopy—liver shift and deformation due to pneumoperitoneum in an animal model. Minim Invasive Ther Allied Technol.

[25] Nickel F, Kenngott HG, Neuhaus J, Sommer CM, Gehrig T, Kolb A, Gondan M, Radeleff BA, Schaible A, Meinzer HP, Gutt CN, Muller-Stich BP (2013). Navigation system for minimally invasive esophagectomy: experimental study in a porcine model. Surg Endosc.

[26] Biolato M, Marrone G, Racco S, Di Stasi C, Miele L, Gasbarrini G, Landolfi R, Grieco A (2010). Transarterial chemoembolization (TACE) for unresectable HCC: a new life begins?. Eur Rev Med Pharmacol Sci.

[27] Lloyd DM, Lau KN, Welsh F, Lee KF, Sherlock DJ, Choti MA, Martinie JB, Iannitti DA (2011). International multicentre prospective study on microwave ablation of liver tumours: preliminary results. HPB (Oxford).

[28] Nickel F, Kenngott HG, Neuhaus J, Andrews N, Garrow C, Kast J, Sommer CM, Gehrig T, Gutt CN, Meinzer HP, Muller-Stich BP (2018). Computer tomographic analysis of organ motion caused by respiration and intraoperative pneumoperitoneum in a porcine model for navigated minimally invasive esophagectomy. Surg Endosc.

[29] Pelanis E, Teatini A, Eigl B, Regensburger A, Alzaga A, Kumar RP, Rudolph T, Aghayan DL, Riediger C, Kvarnström N, Elle OJ, Edwin B (2021). Evaluation of a novel navigation platform for laparoscopic liver surgery with organ deformation compensation using injected fiducials. Med Image Anal.

[30] Sauer IM, Queisner M, Tang P, Moosburner S, Hoepfner O, Horner R, Lohmann R, Pratschke J (2017). Mixed reality in visceral surgery: development of a suitable workflow and evaluation of intraoperative use-cases. Ann Surg.

[31] Cash DM, Sinha TK, Chapman WC, Terawaki H, Dawant BM, Galloway RL, Miga MI (2003). Incorporation of a laser range scanner into image-guided liver surgery: surface acquisition, registration, and tracking. Med Phys.

[32] Mersmann S, Seitel A, Erz M, Jahne B, Nickel F, Mieth M, Mehrabi A, Maier-Hein L (2013). Calibration of time-of-flight cameras for accurate intraoperative surface reconstruction. Med Phys.

[33] Kenngott HG, Wunscher JJ, Wagner M, Preukschas A, Wekerle AL, Neher P, Suwelack S, Speidel S, Nickel F, Oladokun D, Albala L, Maier-Hein L, Dillmann R, Meinzer HP, Muller-Stich BP (2015). OpenHELP (Heidelberg laparoscopy phantom): development of an open-source surgical evaluation and training tool. Surg Endosc.

[34] Wagner M, Gondan M, Zollner C, Wunscher JJ, Nickel F, Albala L, Groch A, Suwelack S, Speidel S, Maier-Hein L, Muller-Stich BP, Kenngott HG (2016). Electromagnetic organ tracking allows for real-time compensation of tissue shift in image-guided laparoscopic rectal surgery: results of a phantom study. Surg Endosc.

[35] Teatini A, Pelanis E, Aghayan D, Kumar RP, Palomar R, Fretland ÅA, Edwin B, Elle OJ (2019). The effect of intraoperative imaging on surgical navigation for laparoscopic liver resection surgery. Sci Rep.

[36] Court FG, Wemyss-Holden SA, Morrison CP, Teague BD, Laws PE, Kew J, Dennison AR, Maddern GJ (2003). Segmental nature of the porcine liver and its potential as a model for experimental partial hepatectomy. Br J Surg.

[37] Schweikard A, Glosser G, Bodduluri M, Murphy MJ, Adler JR (2000). Robotic motion compensation for respiratory movement during radiosurgery. Comput Aided Surg.

[38] Zhang L, Parrini S, Freschi C, Ferrari V, Condino S, Ferrari M, Caramella D (2014). 3D ultrasound centerline tracking of abdominal vessels for endovascular navigation. Int J Comput Assist Radiol Surg.

[39] Srimathveeravalli G, Leger J, Ezell P, Maybody M, Gutta N, Solomon SB (2013). A study of porcine liver motion during respiration for improving targeting in image-guided needle placements. Int J Comput Assist Radiol Surg.

[40] Brix L, Ringgaard S, Sorensen TS, Poulsen PR (2014). Three-dimensional liver motion tracking using real-time two-dimensional MRI. Med Phys.

[41] Narkbuakaew W, Nagahashi H, Aoki K, Kubota Y (2014). Liver segmentation based on reaction-diffusion evolution and Chan–Vese model in 4DCT. Commun Comput Inf Sci.

[42] Vásquez Osorio E, Hoogeman M, Romero A, Wielopolski P, Zolnay A, Heijmen B (2012). Accurate CT/MR vessel-guided nonrigid registration of largely deformed livers. Med Phys.

[43] Mallmann C, Wolf KJ, Wacker FK, Meyer BC (2012). Assessment of patient movement in interventional procedures using electromagnetic detection—comparison between conventional fixation and vacuum mattress. RoFo: Fortschritte auf dem Gebiete der Rontgenstrahlen und der Nuklearmedizin.

[44] Kobr J, Kuntscher V, Treska V, Molacek J, Vobruba V, Fremuth J, Racek J, Trefil L, Kocova J (2008). Adverse effects of the high tidal volume during mechanical ventilation of normal lung in pigs. Bratisl Lek Listy.

[45] Roosens CD, Ama R, Leather HA, Segers P, Sorbara C, Wouters PF, Poelaert JI (2006). Hemodynamic effects of different lung-protective ventilation strategies in closed-chest pigs with normal lungs. Crit Care Med.

[46] Suramo I, Paivansalo M, Myllyla V (1984). Cranio-caudal movements of the liver, pancreas and kidneys in respiration. Acta Radiol Diagn.

[47] Scott J, Singh A, Valverde A (2020). Pneumoperitoneum in veterinary laparoscopy: a review. Vet Sci.

[48] Volz J, Köster S, Weiss M, Schmidt R, Urbaschek R, Melchert F, Albrecht M (1996). Pathophysiologic features of a pneumoperitoneum at laparoscopy: a swine model. Am J Obstet Gynecol.

[49] Maracaja-Neto LF, Vercosa N, Roncally AC, Giannella A, Bozza FA, Lessa MA (2009). Beneficial effects of high positive end-expiratory pressure in lung respiratory mechanics during laparoscopic surgery. Acta Anaesthesiol Scand.

[50] Nguyen NT, Anderson JT, Budd M, Fleming NW, Ho HS, Jahr J, Stevens CM, Wolfe BM (2004). Effects of pneumoperitoneum on intraoperative pulmonary mechanics and gas exchange during laparoscopic gastric bypass. Surg Endosc.

[51] Oikkonen M, Tallgren M (1995). Changes in respiratory compliance at laparoscopy: measurements using side stream spirometry. Can J Anaesth.

[52] Fahy BG, Barnas GM, Nagle SE, Flowers JL, Njoku MJ, Agarwal M (1996). Changes in lung and chest wall properties with abdominal insufflation of carbon dioxide are immediately reversible. Anesth Analg.

[53] Chang KH, Ho MC, Yeh CC, Chen YC, Lian FL, Lin WL, Yen JY, Chen YY (2012). Effectiveness of external respiratory surrogates for in vivo liver motion estimation. Med Phys.

[54] Herline AJ, Stefansic JD, Debelak JP, Hartmann SL, Pinson CW, Galloway RL, Chapman WC (1999). Image-guided surgery: preliminary feasibility studies of frameless stereotactic liver surgery. Arch Surg.

[55] Vijayan S, Reinertsen I, Hofstad EF, Rethy A, Hernes TA, Lango T (2014). Liver deformation in an animal model due to pneumoperitoneum assessed by a vessel-based deformable registration. Minim Invasive Ther Allied Technol.

[56] Heizmann O, Zidowitz S, Bourquain H, Potthast S, Peitgen HO, Oertli D, Kettelhack C (2010). Assessment of intraoperative liver deformation during hepatic resection: prospective clinical study. World J Surg.

[57] Lesage D, Angelini E, Bloch I, Funka-Lea G (2009) A review of 3D vessel lumen segmentation techniques: models, features and extraction schemes. Med Image Anal 13:819–845 10.1016/j.media.2009.07.011 19818675

[58] Beddar AS, Kainz K, Briere TM, Tsunashima Y, Pan T, Prado K, Mohan R, Gillin M, Krishnan S (2007) Correlation between internal fiducial tumor motion and external marker motion for liver tumors imaged with 4D-CT. Int J Radiat Oncol Biol Phys 67:630–638. 10.1016/j.ijrobp.2006.10.007 10.1016/j.ijrobp.2006.10.007 17236980

[59] Xi M, Liu MZ, Li QQ, Cai L, Zhang L, Hu YH (2009) Analysis of abdominal organ motion using four-dimensional CT. Ai Zheng 28:989–993 10.5732/cjc.009.10193 19728920

[60] Nguyen TN, Moseley JL, Dawson LA, Jaffray DA, Brock KK (2009) Adapting liver motion models using a navigator channel technique. Med Phys 36:1061–1073 10.1118/1.3077923 PMC2736751 19472611

